# Influence of age on androgen deprivation therapy-associated Alzheimer’s disease

**DOI:** 10.1038/srep35695

**Published:** 2016-10-18

**Authors:** Kevin T. Nead, Greg Gaskin, Cariad Chester, Samuel Swisher-McClure, Joel T. Dudley, Nicholas J. Leeper, Nigam H. Shah

**Affiliations:** 1 Stanford Center for Biomedical Informatics Research, Stanford University, 1265 Welch Road, Stanford, California, 94305, USA; 2 Department of Radiation Oncology, University of Pennsylvania Perelman School of Medicine, 3400 Civic Center Boulevard, Philadelphia, Pennsylvania, 19104, USA; 3 Department of Population Health Science and Policy, Icahn School of Medicine at Mount Sinai, One Gustave L. Levy Place, New York, 10029, USA; 4 Stanford Cardiovascular Institute, Stanford University School of Medicine, 300 Pasteur Drive, Stanford, California, 94305, USA.

## Abstract

We recently found an association between androgen deprivation therapy (ADT) and Alzheimer’s disease. As Alzheimer’s disease is a disease of advanced age, we hypothesize that older individuals on ADT may be at greatest risk. We conducted a retrospective multi-institutional analysis among 16,888 individuals with prostate cancer using an informatics approach. We tested the effect of ADT on Alzheimer’s disease using Kaplan–Meier age stratified analyses in a propensity score matched cohort. We found a lower cumulative probability of remaining Alzheimer’s disease-free between non-ADT users age ≥70 versus those age <70 years (p < 0.001) and between ADT versus non-ADT users ≥70 years (p = 0.034). The 5-year probability of developing Alzheimer’s disease was 2.9%, 1.9% and 0.5% among ADT users ≥70, non-ADT users ≥70 and individuals <70 years, respectively. Compared to younger individuals older men on ADT may have the greatest absolute Alzheimer’s disease risk. Future work should investigate the ADT Alzheimer’s disease association in advanced age populations given the greater potential clinical impact.

Prostate cancer is diagnosed in over 1 million patients each year^1^. Androgen deprivation therapy (ADT) is a mainstay of treatment for men with unfavorable and advanced prostate cancer^2^ with over 50% of prostate cancer patients in industrialized nations utilizing ADT^3^. Importantly, ADT has been associated with both improved overall survival and increased adverse health effects^4^,^5^ with emerging data indicating a detrimental impact on neurocognitive function^6^.

We previously demonstrated an association between ADT and Alzheimer’s disease^7^. In this study we found a large relative increased risk of Alzheimer’s disease among ADT users. Currently, it is unclear among which groups this association may have the greatest clinical significance^8^. Given that Alzheimer’s disease is a disease of older age, and that ADT is unlikely sufficient to cause Alzheimer’s disease in isolation, we hypothesize that ADT may have a larger absolute impact on Alzheimer’s disease risk among older patients. Here we utilize an informatics approach to analyze electronic medical data in over 5 million patients to examine the impact of age on the association of ADT with Alzheimer’s disease.

## Materials and Methods

We used a validated text-processing pipeline to analyze electronic medical record data at Stanford University and Mount Sinai hospitals with study characteristics previously described^7^. Both data sources were accessed under approved institutional review board protocols. Access to Mt. Sinai data was obtained via an institutional research agreement. The institutional review board waived the requirement for patient consent as the data mining studies were deemed not to involve human participants.

Briefly, individuals with prostate cancer and follow-up ≥180 days after diagnosis were eligible. Prostate cancer was defined as (1) ICD-9 code (185), (2) billing code for radical prostatectomy (ICD-9 60.5 or CPT code 55810–55815, 55840–55845) plus either ADT use (in medication lists or clinical text) or clinical text evidence of prostate cancer diagnosis, or (3) clinical text evidence of prostate cancer diagnosis and ADT use (in medication lists or clinical text). The use of ADT was defined using data from clinical notes and medication lists including pharmacy orders.

Those with a history of dementia, stroke or chemotherapy use were excluded. Covariates were age at prostate cancer diagnosis, race, smoking status, use of anti-platelet, anti-coagulant, anti-hypertensive and statin medications, and a history of cardiovascular disease, diabetes or malignancy. Variables were defined using ICD-9 diagnostic codes, CPT codes, medications lists and clinical text^7^.

We examined the impact of ADT on Alzheimer’s disease stratified by age at diagnosis. Age 70 years was selected as the cut-off given existing management guidelines for cancer patients older than 70 regarding assessment for age appropriate intervention^9^. There was insufficient power to examine the association of ADT and Alzheimer’s disease in the <70 years subgroup given 9,112 non-ADT users with 35 Alzheimer’s disease cases and 1,105 ADT users with 3 Alzheimer’s disease cases, which allows a detectable hazard ratio (HR) ≥4.3.

Patient characteristics were compared using a t-test or chi-squared test. Hazard ratios were calculated using 1:5 propensity score matched and traditional multivariable adjusted Cox proportional hazards models to test the effect of use versus non-use of ADT, and ADT duration (no-ADT, <12 months, ≥12 months), on risk of Alzheimer’s disease. Proportional-hazards assumptions were evaluated by Schoenfeld’s residuals tests. Kaplan–Meier curves were compared among individuals <70 years, non-ADT users ≥70 years, and ADT users ≥70 years in the full and propensity score matched cohorts using log-rank and Cox tests for equality, respectively. A test for interaction was conducted between age and ADT using the Wald test. We additionally calculated the cumulative probability of developing Alzheimer’s disease at 5-years using the Kaplan-Meier method in the age-stratified propensity score matched cohort.

A 2-sided p-value < 0.05 was considered significant. Analyses were performed using Stata version 12.0 (StataCorp, College Station, TX) and R version 3.2 (R Foundation for Statistical Computing, Vienna, Austria).

## Results

Baseline patient characteristics are shown in Table 1. No statistically significant differences existed between ADT and non-ADT users ≥70 years in the propensity score matched cohort. The median follow-up period was 2.7 years (interquartile range [IQR], 1.0–5.4 years). The median time to the diagnosis of Alzheimer’s disease was 4.0 years (IQR, 2.0 to 7.4 years).

**Figure 1:**
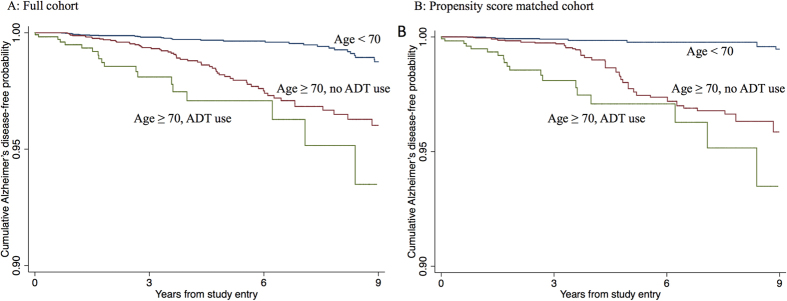
Kaplan-Meier curves according to androgen deprivation therapy (ADT) status and age for the cumulative probability of remaining Alzheimer’s disease-free (y-axis) from the initiation of ADT, for ADT users, or from the time of prostate cancer diagnosis plus the median time to ADT use, for non-ADT users (x-axis) in the full (**A**) Age <70 versus Age ≥70, p < 0.001; Age < 70 versus Age ≥70 without ADT use, p < 0.001; Age ≥70 with ADT use versus Age ≥70 without ADT use, p = 0.008) and propensity score matched cohorts (**B**) Age <70 versus Age ≥70, p < 0.001; Age <70 versus Age ≥70 without ADT use, p < 0.001; Age ≥70 with ADT use versus Age ≥70 without ADT use, p = 0.034) AD, Alzheimer’s disease; ADT, androgen deprivation therapy.

Kaplan-Meier curves (Fig. 1) demonstrated a lower cumulative probability of remaining Alzheimer’s disease-free between those age ≥70 years without ADT use versus those age <70 years (p < 0.001) and between ADT users ≥70 years versus non-ADT users ≥70 years (p = 0.034) in the propensity score matched cohort. The cumulative probability of developing Alzheimer’s disease at 5-years was 2.9%, 1.9% and 0.5% among ADT users ≥70 years, non-ADT users ≥70 years and individuals <70 years of age, respectively. There was a statistically significant association between ADT use and Alzheimer’s disease among those ≥70 years using propensity score matched (HR = 1.84; 95% confidence interval [CI], 1.07–3.17; p = 0.027) and traditional multivariable adjusted Cox regression analysis (HR = 2.04; 95% CI, 1.23–3.40; p = 0.006). Among individuals ≥70 years, a longer duration of ADT was associated with a greater risk of Alzheimer’s disease (HR = 1.41; 95% CI 1.01–1.96, p = 0.043). We did not find evidence of an interaction between ADT use and age (Wald 0.08, p = 0.782).

## Conclusions

Using an informatics approach we find that, compared to younger individuals, men aged 70 years or older on ADT have a clinically significant increase in absolute Alzheimer’s disease risk. We support this finding using both multivariable adjusted and propensity score matched models in a large cohort of individuals. This further supports the association between ADT and cognitive dysfunction and suggests that older men may be most susceptible and a particular high-risk subgroup deserving further investigation^6^,^7^.

Multiple studies now demonstrate an association between ADT and neurocognitive dysfunction^6^,^7^,^10^. The association of ADT and Alzheimer’s disease is supported by a number of plausible biologic mechanisms including through augmentation of β-amyloid protein levels^11^, interaction with the Apolipoprotein E gene^12^, a direct neuropathic effect^13^ and an increase in cardiometabolic disease^5^,^14^. If ADT is truly causally associated with Alzheimer’s disease it likely contributes within a multifactorial etiology. Given that age is the strongest risk factor for Alzheimer’s disease^15^ we therefore postulated that ADT use among older individuals might confer the greatest absolute risk of Alzheimer’s disease, as has previously been found regarding the impact of ADT on cardiovascular disease risk^16^. Our finding that older men on ADT have a greater absolute risk of Alzheimer’s disease compared to younger individuals is particularly relevant given concerns regarding aggressive treatment of prostate cancer among men with limited life expectancies^17^.

Limitations of this study include its retrospective design and the inability to conduct subgroup analysis according to ADT use versus non-use in the <70 years cohort due to low event rates. We were unable to account for prostate cancer specific characteristics, such as Gleason score. We were not powered to undertake subgroup analysis by type of ADT, which may be relevant given that some types of ADT might have a protective effect on Alzheimer’s disease^18^. Finally, we were unable to evaluate APOE ε4 allele status, which may interact with testosterone levels^19^.

In conclusion, we find that older men on ADT have the greatest absolute increased risk of Alzheimer’s disease. Future studies are required to determine the mechanism of this association and to develop preventative strategies and inform clinical practice. Prioritization of research and clinical intervention regarding adverse effects of ADT among older individuals may have the greatest clinical impact.

## Additional Information

**How to cite this article**: Nead, K. T. *et al*. Influence of age on androgen deprivation therapy-associated Alzheimer’s disease. *Sci. Rep.* **6**, 35695; doi: 10.1038/srep35695 (2016).

## Figures and Tables

**Table 1:** Baseline patient characteristics in the full and propensity score matched cohorts.

	Full cohort						Propensity score matched cohort					
	All age-groups			Age ≥70 years subgroup			All age-groups			Age ≥70 years subgroup		
	ADT (n = 2,397)	No ADT (n = 14,491)	p-value	ADT (n = 1,292)	No ADT (n = 5,379)	p-value	ADT (n = 2,397)	No ADT (n = 11,985)	p-value	ADT (n = 1,292)	No ADT (n = 6,339)	p-value
Characteristic												
Age, mean years (SD)	70.9 (10.8)	66.7 (10.5)	<0.001	78.9 (6.9)	77.5 (6.5)	<0.001	70.9 (10.8)	70.9 (12.6)	0.974	78.9 (6.9)	78.9 (8.4)	0.902
Caucasian	1243 (52)	8426 (58)	<0.001	678 (52)	3,249 (60)	<0.001	1243 (52)	6,487 (54)	0.115	678 (52)	3,482 (55)	0.213
Ever smoker	890 (37)	3420 (24)	<0.001	461 (36)	1,308 (24)	<0.001	890 (37)	4,553 (38)	0.539	461 (36)	2,353 (37)	0.450
Anti-platelet use	802 (33)	3394 (23)	<0.001	515 (40)	1,761 (33)	<0.001	802 (33)	3,871 (32)	0.393	515 (40)	2,441 (39)	0.483
Anti-coagulant use	420 (18)	1885 (13)	<0.001	291 (23)	1,085 (20)	0.061	420 (18)	1,950 (16)	0.248	291 (23)	1,388 (22)	0.703
Anti-hypertensive use	1205 (50)	5775 (40)	<0.001	738 (57)	2,693 (50)	<0.001	1205 (50)	6,015 (50)	0.954	738 (57)	3,644 (58)	0.852
Statin use	559 (23)	3135 (22)	0.064	355 (28)	1,512 (28)	0.649	559 (23)	2,651 (22)	0.321	355 (28)	1,690 (27)	0.642
Cardiovascular disease	679 (28)	3072 (21)	<0.001	483 (37)	1,885 (35)	0.114	679 (28)	3,288 (27)	0.491	483 (37)	2,422 (38)	0.667
Diabetes	514 (21)	2295 (16)	<0.001	282 (22)	1,045 (19)	0.052	514 (21)	2,499 (21)	0.616	282 (22)	1,408 (22)	0.814
Malignancy	166 (7)	1057 (7)	0.519	111 (9)	571 (11)	0.031	166 (7)	679 (6)	0.073	111 (9)	460 (7)	0.212

ADT, Androgen deprivation therapy; SD, standard deviation.

All data reported as number (%) unless otherwise noted.
